# Association of hyperglycemia in a general Japanese population with late-night-dinner eating alone, but not breakfast skipping alone

**DOI:** 10.1186/s40200-015-0147-0

**Published:** 2015-03-25

**Authors:** Kei Nakajima, Kaname Suwa

**Affiliations:** Division of Clinical Nutrition, Department of Medical Dietetics, Faculty of Pharmaceutical Sciences, Josai University, 1-1 Keyakidai, Sakado, Saitama 350-0295 Japan; Saitama Health Promotion Corporation, Saitama, Japan

**Keywords:** Late-night-dinner eating, Breakfast skipping, Hyperglycemia, Diabetes, Unhealthy eating habit, HbA1c, Obesity

## Abstract

The unhealthy habit of late-night-dinner eating (LNDE) is often observed in adults. Since LNDE can lead to breakfast skipping (BS) the next morning, we examined the associations of LNDE and BS with hyperglycemia (HbA1c ≥ 5.7% and/or pharmacotherapy for diabetes), separately and in combination, in 61,364 apparently healthy Japanese adults aged 30–70 years. Although LNDE alone was significantly associated with hyperglycemia, even after adjustment for body mass index, BS alone was not. Our results indicate that hyperglycemia in the general Japanese population is associated with LNDE alone, but not BS alone.

Although the prevalence of obesity in Japan and other Asian countries remains lower than in Western countries, that of type 2 diabetes is rising [1]. Among the various lifestyle factors that affect the onset of obesity and type 2 diabetes, is the unhealthy eating habit of breakfast skipping (BS) [2-5]. Owing to the variety of concomitant confounding factors however, including nocturnal life, impaired appetite and satiety, smoking, reduced physical activity and frequent alcohol drinking [3,4,6], the mechanisms underlying this association are poorly understood. Moreover, the lack of a global concept and appropriate worldwide definition of BS may obscure its associations with other unhealthy eating habits and cardiometabolic conditions.

Owing to a variety of factors, including lack of time, impaired appetite, and fatigue, the incidence of late-night dinner eating (LNDE) can be associated with that of BS the following morning. We have previously shown that LNDE plus BS is significantly associated with metabolic syndrome, proteinuria, and incidence of atrial fibrillation [6,7], and that BS alone in subjects not undergoing pharmacotherapy for diabetes is not associated with high-normal HbA_1c_ (≥5.6%) [6]. Here we studied the separate associations of habitual LNDE and BS with hyperglycemia in a general Japanese population.

## Methods

### Subjects

This cross-sectional study consisted of data recorded during annual medical checkups of asymptomatic people living or working in Saitama Prefecture, a suburb of Tokyo, Japan [8]. We reviewed the data for 102,046 subjects who underwent a medical health check-up at the Saitama Health Promotion Corporation in 2012. After age restriction and exclusion of subjects with incomplete data, 61,364 apparently healthy subjects (36,416 men and 24,948 women), aged 30–70 years, were enrolled. In our previous studies, where the age of subjects ranged from 20 to 75 or 80 years old, the prevalence of hyperglycemia was very low in the younger age group and the prevalence of BS was very low in the older age group [6,9]. Accordingly, in this study, we restricted subjects to those aged 30–70 years.

### Measurements

Anthropometric and laboratory tests were carried out in the morning after overnight fasting. Serum parameters were measured using standard methods at the Saitama Health Promotion Corporation. HbA_1c_ (Japan Diabetes Society [JDS]) was converted to HbA_1c_ (National Glycohemoglobin Standardization Program [NGSP]) units using the officially certified formula: HbA_1c_ (NGSP) (%) = 1.02 × JDS (%) + 0.25% [10].

Questions for detecting unhealthy eating habits were developed by the Japanese Ministry of Health, Labour and Welfare in 2008 [8,11]. Habitual BS and LNDE were determined based on a positive response to the question: “Do you skip breakfast at least three times per week?” and “Do you eat dinner within 2 hours before bedtime at least three times per week?”, respectively. In this study, LNDE did not necessarily mean that dinner was eaten particularly late (e.g., around midnight) but rather that dinner was eaten shortly before bedtime. In addition, we took into consideration the question about the sleep and sufficient rest because LNDE can deteriorate the quality and the duration of sleep. Self-reported sufficient rest from sleep was determined based on a positive response to the question: “Do you get sufficient rest from sleep?” According to the combination of LNDE and BS, subjects were classified into four eating habit groups: absence of LNDE and BS (normal eating habit), LNDE alone, BS alone, and LNDE plus BS. Hyperglycemia was defined as HbA_1c_ ≥ 5.7% and/or pharmacotherapy for diabetes [12].

### Statistical analysis

Data are expressed as mean ± standard deviation. Continuous and categorical variables between the four eating habit groups were examined by analysis of variance (ANOVA) and χ^2^-test, respectively. Post hoc test by Tukey-Kramer test and additional χ^2^-test were used to examine the difference between specific two groups. Multivariate logistic regression models were used to examine the associations of hyperglycemia with three unhealthy eating habit groups. Logistic regression models yielded ORs and 95% CIs. Statistical analyses were performed using Statview version 5.0 (SAS Institute; Cary, NC, USA). Values of *p* < 0.05 were considered statistically significant.

### Ethics approval

The protocol was approved by the Ethics Committees of Josai University and Jichi Medical University, and the committee of the Saitama Health Promotion Corporation, a public interest corporation. Written informed consent was obtained from all participants.

## Results

The characteristics of the subjects are shown in Table 1. LNDE alone was more prevalent than BS alone and LNDE plus BS in all age groups. The prevalence of BS alone was comparable to that of LNDE plus BS, indicating that half of BS accompanies LNDE, particularly in younger age groups. Compared with subjects with normal eating habits, individuals who reported LNDE or BS were more likely to be men, daily alcohol drinkers, current smokers, infrequent exercisers, to have less self-reported sufficient rest from sleep, and to have greater cardiovascular risk factors, including higher BMI and waist circumference (all p values by ANOVA and χ^2^-test were < 0.0001). They were also more likely to be younger and to have lower HbA_1c_. Approximately one-fifth of observed hyperglycemia corresponded to overt diabetes (HbA1c ≥ 6.5%).

**Table 1:** **Clinical characteristics of subjects according to the four eating habits**

**Eating-habit groups**	**Absence of LNDE and BS**	**LNDE alone**	**BS**		**Post hoc test**
			**BS alone**	**LNDE plus BS**	
*N* (% of total)	38,475 (62.7)	11,718 (19.1)	5880 (9.6)	5291 (8.6)	
30–39 years (%)^*^	52.7	21.6	13.0	12.7	
40–49 years (%)^*^	58.8	20.6	10.5	10.2	
50–59 years (%)^*^	67.0	18.6	7.9	6.5	
60–70 years (%)^*^	77.3	14.1	5.5	3.1	
Men, n (%)	20,611 (53.6)	7883 (67.3)	3948 (67.1)	3974 (75.1)	a,b,c,e,f
Age (years)	49.6 ± 11.4	46.3 ± 10.8	44.5 ± 10.5	43.0 ± 9.6	a,b,c,d,e,f
BMI (kg/m^2^)	23.1 ± 3.5	23.7 ± 3.7	23.5 ± 3.8	23.7 ± 3.9	a,b,c,d,f
Waist circumference (cm)	81.6 ± 9.5	83.0 ± 10.0	82.1 ± 10.1	82.9 ± 10.3	a,b,c,d,f
Systolic blood pressure (mmHg)	124 ± 16.9	125 ± 17.0	124 ± 16.9	125 ± 16.0	a,c
Diastolic blood pressure (mmHg)	75 ± 12	76 ± 12	76 ± 13	76 ± 13	a,b,c
Triglycerides (mg/dl)	93 (64–140)	95 (64–148)	98 (67–153)	98 (65–155)	a,b,c,e
HDL-cholesterol (mg/dl)	59.7 ± 14.8	58.9 ± 14.8	57.3 ± 14.9	57.4 ± 15.1	a,b,c,d,e
HbA1c (%, NGSP)	5.63 ± 0.68	5.62 ± 0.74	5.58 ± 0.73	5.57 ± 0.73	b,c,d,e
Hyperglycemia, n (%)	10,844 (28.2)	3080 (26.3)	1324 (22.5)	1165 (22.0)	a,b,c,d,e
Pharmacotherapy for					
Hypertension, n (%)	5300 (13.8)	1424 (12.2)	465 (7.9)	374 (7.1)	a,b,c,d,e
Diabetes, n (%)	1365 (3.5)	403 (3.4)	107 (1.8)	98 (1.9)	b,c,d,e
Dyslipidemia, n (%)	3066 (8.0)	667 (5.7)	236 (4.0)	187 (3.5)	a,b,c,d,e
Past history of CVD, n (%)	996 (2.6)	301 (2.6)	106 (1.8)	87 (1.6)	b,c,d,e
Daily alcohol consumption, n (%)	8067 (21.0)	4273 (36.5)	1354 (23.0)	1855 (35.1)	a,b,c,d,f
Current smoker, n (%)	7801 (20.3)	3560 (30.4)	2832 (48.2)	2861 (54.1)	a,b,c,d,e,f
Having regular exercise, n (%)	11,022 (28.6)	2741 (23.4)	1237 (21.0)	1020 (19.3)	a,b,c,d,e
Self-reported full-rest by sleep, n (%)	25,580 (66.5)	5936 (50.7)	3193 (54.3)	2190 (41.4)	a,b,c,d,e,f

Data are expressed as mean ± SD. Triglyceride is expressed as median (interquartile range).

Continuous and categorical variables between the four eating habit groups were examined by ANOVA and χ^2^ tests.

All *p* values by ANOVA and χ^2^ tests were < 0.0001.

Post hoc test by Tukey-Kramer test was used to examine the difference between specific two groups. Additional χ^2^-test was conducted to examine the difference between specific two groups (p < 0.0071 was considered statistically significant).

^a^Absence of LNDE and BS vs LNDE alone.

^b^Absence of LNDE and BS vs BS alone. ^c^Absence of LNDE and BS vs LNDE plus BS.

^d^LNDE alone vs BS alone.

^e^LNDE alone vs LNDE plus BS.

^f^BS alone vs LNDE plus BS.

^*^Percentage of each age group.

BMI, body mass index, BS, breakfast skipping; CVD, cardiovascular disease (including stroke); LNDE, late-night-dinner eating; NGSP, National Glycohemoglobin Standardization Program.

Table 2 shows the results of multivariate logistic regression analysis. After adjustment for confounders including age and sex, LNDE alone and LNDE plus BS were significantly associated with hyperglycemia (Model 3). Although the association between LNDE plus BS and hyperglycemia disappeared after further adjustment for BMI, the association between LNDE alone and hyperglycemia remained significant even after adjustment for BMI and self-reported sufficient rest from sleep (Model 4 and Model 5). In contrast, BS alone was persistently found not to be associated with hyperglycemia. It is noteworthy that BS alone was, if anything, inversely associated with hyperglycemia (OR 0.94; 95% CI 0.87, 1.01; *p* = 0.08, Model 5).

**Table 2:** **Odds ratios and 95% CIs of unhealthy eating habits for hyperglycemia**

	**Absence of LNDE and BS**	**LNDE alone**	**BS alone**	**LNDE plus BS**
Hyperglycemia				
Model 1	1 (Ref)	0.91 (0.87–0.95)^b^	0.74 (0.69–0.79)^b^	0.72 (0.67–0.77)^b^
Model 2	1 (Ref)	1.13 (1.07–1.18)^b^	1.03 (0.96–1.11)	1.12 (1.04–1.21)^a^
Model 3	1 (Ref)	1.21 (1.15–1.27)^b^	0.99 (0.92–1.06)	1.12 (1.04–1.21)^a^
Model 4	1 (Ref)	1.13 (1.07–1.19)^b^	0.94 (0.88–1.01)	1.05 (0.97–1.13)
Model 5	1 (Ref)	1.12 (1.06–1.18)^b^	0.94 (0.87–1.01)	1.03 (0.95–1.11)

^a^ *p* < 0.01 ^b^ *p* < 0.0001.

Hyperglycemia was defined as HbA_1c_ ≥5.7% and/or pharmacotherapy for diabetes.

Model 1: Unadjusted.

Model 2: Adjusted for age and sex.

Model 3: Model 2 plus current smoking, daily alcohol consumption, regular exercise, pharmacotherapy for hypertension and dyslipidemia, and history of cardiovascular disease.

Model 4: Model 3 plus body mass index (as a continuous variable).

Model 5: Model 4 plus self-reported full rest from sleep (presence vs absence).

All three eating habit categories (LNDE alone, BS alone, and LNDE plus BS) were significantly associated with obesity (BMI ≥ 25 kg/m^2^) after adjustment for all confounders in Model 5 except continuous BMI (OR 1.28, 95% CI 1.22, 1.35; OR 1.21, 95% CI 1.13, 1.29; and OR 1.26, 95% CI 1.18, 1.35, respectively; all *p* < 0.0001).

When subjects were restricted to a subgroup without overt diabetes (HbA1c < 6.5%, n = 58,117) and the same analysis was conducted, similar results were observed.

## Discussion

Our study demonstrated that hyperglycemia in a general Japanese population was robustly associated with LNDE alone, independent of relevant confounders including BMI, but not with BS alone. The association between LNDE plus BS and hyperglycemia was dependent on BMI. We observed similar findings in our previous study [6] of other Japanese subjects without pharmacotherapy for diabetes who underwent a check-up in 2008. Similar to the current study, in the previous study BS alone was not significantly associated with high-normal HbA_1c_ (≥5.6%). Unlike the current study however, a significant association between LNDE alone and high-normal HbA_1c_ was not observed after further adjustment for BMI in the previous study. A plausible explanation for this discrepancy is that in the current study, impaired glucose metabolism was evaluated by hyperglycemia defined as HbA_1c_ ≥5.7% and/or pharmacotherapy for diabetes, instead of high-normal HbA_1c_ alone. In addition, the restriction of subjects to those without pharmacotherapy for diabetes and wider age range (20–75 years) in the previous study [6] might generate a bias in the outcomes between previous study and current study.

Although an association between BS and obesity and type 2 diabetes has been indicated by several studies encompassing confounding factors such as smoking, alcohol drinking, and physical activity [2-5], these reports did not account for unhealthy eating habits, such as LNDE, that likely elicit the incident of BS. In our previous and current studies [6,7], approximately 50% of subjects reporting BS also reported LNDE, an association that was maintained in all age groups (Table 1). Moreover, the prevalence of LNDE was higher than that of BS, suggesting that compared with BS, LNDE is a significant public health issue. Taken together, our series of studies suggests that BS and LNDE should be evaluated both separately and in combination in the assessment of cardiometabolic conditions.

As with the other two eating habits (LNDE alone and LNDE plus BS), BS alone was significantly associated with obesity, a well-documented risk factor for type 2 diabetes, in this study. Despite this, BS alone was not associated with hyperglycemia. Similarly, Odegaard et al. [5] reported that frequent BS in black women was significantly associated with obesity and metabolic syndrome, but not type 2 diabetes, suggesting that the impact of BS on hyperglycemia may vary according to sex and race.

The evolution of BS alone into concomitant BS and LNDE over time may be attributable to up-regulated appetite later in the day [3], resulting in a progressively worsening health condition. Conversely, LNDE alone can sometimes accompany BS, probably owing to a lack of sufficient time to eat or reduced hunger in the morning, which may be consistent with the smaller portion of self-reported full-rest by sleep in subjects with LNDE plus BS (Table 1). However, not eating breakfast might protect against such deterioration of glucose metabolism. Theoretically, LNDE, particularly soon before sleep, may prolong the postprandial glucose spike for a long time [13] owing to a variety of mechanisms such as the lack of physical activity during sleep. When breakfast is subsequently eaten without an adequate interval, the restoration of elevated plasma glucose to normal levels may be further hindered. Although promoting the eating of breakfast may be beneficial in the overall population, recommending this habit in individuals who habitually eat dinner late at night, particularly shortly before sleep, requires prior consideration of LNDE and nocturnal life as a primary target in ameliorating their cardiometabolic status.

Several limitations should be mentioned in the current study. First, this study was cross-sectional in nature, and did not allow us to determine the causality between unhealthy eating-habits and lifestyle such as smoking and alcohol drinking and hyperglycemia. Second, assessment of food intake, for instance, using food-frequency questionnaires, is required to consider the quality of diet in subjects. In this context, Odegaard et al. [5] have shown that the association between breakfast intake and reduced metabolic conditions was not related to the overall quality of the dietary pattern. In addition, considering the large sample size in this study of a general population, a detailed assessment of dietary composition may not be feasible because of time and cost restrictions. Finally, patients with overt diabetes are commonly treated with medications, which can substantially affect the appetite and food consumption. Therefore, when the association of unhealthy eating habits with moderate to sever diabetes is examined, different results might be observed.

## Conclusions

In conclusion, we have found that hyperglycemia in a general Japanese adult population is associated with LNDE alone, but not BS alone. These results suggest the possibility that eating a full breakfast particularly rich in carbohydrate should be avoided for the prevention of diabetes in individuals with unhealthy nocturnal life including habitual LNDE. However, further prospective and clinical trial studies will be needed to confirm the current observations.

## Abbreviations

ANOVA: Analysis of variance
BMI: Body mass index
BS: Breakfast skipping
CI: Confidence interval
LNDE: Late-night dinner eating
NGSP: National glycohemoglobin standardization program
OR: Odds ratio

## References

[1] Yoon KH, Lee JH, Kim JW, Cho JH, Choi YH, Ko SH (2006). Epidemic obesity and type 2 diabetes in Asia. Lancet.

[2] Horikawa C, Kodama S, Yachi Y, Heianza Y, Hirasawa R, Ibe Y (2011). Skipping breakfast and prevalence of overweight and obesity in Asian and Pacific regions: a meta-analysis. Prev Med.

[3] Pereira MA, Erickson E, McKee P, Schrankler K, Raatz SK, Lytle LA (2011). Breakfast frequency and quality may affect glycemia and appetite in adults and children. J Nutr.

[4] Reutrakul S, Hood MM, Crowley SJ, Morgan MK, Teodori M, Knutson KL (2014). The relationship between breakfast skipping, chronotype, and glycemic control in type 2 diabetes. Chronobiol Int.

[5] Odegaard AO, Jacobs DR, Steffen LM, Van Horn L, Ludwig DS, Pereira MA (2013). Breakfast frequency and development of metabolic risk. Diabetes Care.

[6] Kutsuma A, Nakajima K, Suwa K (2014). Potential association between breakfast skipping and concomitant late-night-dinner eating with metabolic syndrome and proteinuria in the Japanese population. Scientifica.

[7] Nakajima K, Suwa K, Oda E (2014). Atrial fibrillation may be prevalent in individuals who report late-night dinner eating and concomitant breakfast skipping, a complex abnormal eating behavior around sleep. Int J Cardiol.

[8] Muneyuki T, Suwa K, Oshida H, Takaoka T, Kutsuma A, Yoshida T (2013). Design of the Saitama cardiometabolic disease and organ impairment study (SCDOIS): a multidisciplinary observational epidemiological study. Open J Endocr Metab Dis.

[9] Oshida H, Kutsuma A, Nakajima K (2013). Associations of eating a late-evening meal before bedtime with low serum amylase and unhealthy conditions. J Diabetes Metab Disord.

[10] Kashiwagi A, Kasuga M, Araki E, Oka Y, Hanafusa T, Ito H (2012). Committee on the standardization of diabetes mellitus-related laboratory testing of Japan diabetes society. International clinical harmonization of glycated hemoglobin in Japan: from Japan diabetes society to national glycohemoglobin standardization program values. J Diabetes Investig.

[11] Ministry of Health, Labour, and Welfare, Health. Report 2008-2009 No. 7 Specific Health Checkups and Specific Health Guidance. http://www.mhlw.go.jp/english/wp/wp-hw3/02.html.

[12] American Diabetes Association (2013). Standards of medical care in diabetes-2013. Diabetes Care.

[13] Sato M, Nakamura K, Ogata H, Miyashita A, Nagasaka S, Omi N (2011). Acute effect of late evening meal on diurnal variation of blood glucose and energy metabolism. Obes Res Clin Pract..

